# Defects in multiple complexes of the respiratory chain are present in ageing human colonic crypts

**DOI:** 10.1016/j.exger.2010.01.013

**Published:** 2010-08

**Authors:** Laura C. Greaves, Martin J. Barron, Stefan Plusa, Thomas B. Kirkwood, John C. Mathers, Robert W. Taylor, Doug M. Turnbull

**Affiliations:** a Mitochondrial Research Group, Institute for Ageing and Health, Medical School, University of Newcastle upon Tyne, Framlington Place, Newcastle upon Tyne, NE2 4HH, UK; b Department of Surgery, Medical School, University of Newcastle upon Tyne, Framlington Place, Newcastle upon Tyne, NE2 4HH, UK; c Institute for Ageing and Health, Henry Wellcome Laboratory for Biogerontology Research, Newcastle University, Campus for Ageing and Vitality, Newcastle upon Tyne, NE4 5PL, UK; d Human Nutrition Research Centre, Institute for Ageing and Health, Medical School, University of Newcastle upon Tyne, Framlington Place, Newcastle upon Tyne, NE2 4HH, UK

**Keywords:** Ageing, Mitochondria, mtDNA, Colon, Respiratory chain, Mutation

## Abstract

Mitochondrial DNA (mtDNA) mutations accumulate in a number of ageing tissues and are proposed to play a role in the ageing process. We have previously shown that colonic crypt stem cells accumulate somatic mtDNA point mutations during ageing. These mtDNA mutations result in the loss of the activity of complex IV (cytochrome *c* oxidase (COX)) of the respiratory chain in the stem cells and their progeny, producing colonic crypts which are entirely COX deficient. However it is not known whether the other complexes of the respiratory chain are similarly affected during ageing. Here we have used antibodies to individual subunits of complexes I–IV to investigate their expression in the colonic epithelium from human subjects aged 18–84. We show that in ∼50% of crypts with any form of respiratory chain deficiency, decreased expression of subunits of multiple complexes is observed. Furthermore we have sequenced the entire mitochondrial genome of a number of cells with multiple complex defects and have found a wide variety of point mutations in these cells affecting a number of different protein encoding and RNA encoding genes. Finally we discuss the possible mechanisms by which multiple respiratory chain complex defects may occur in these cells.

## 1 Introduction

Mitochondria are the energy transducing organelles of eukaryotic cells in which fuels to drive cellular metabolism are converted to ATP. This occurs through the process of oxidative phosphorylation which is catalyzed by the four enzyme complexes of the respiratory chain (NADH-ubiquinone oxidoreductase (complex I), succinate-ubiquinone oxidoreductase (complex II), ubiquinol cytochrome *c* oxidoreductase (complex III), cytochrome *c* oxidase (complex IV)) and the ATP synthase (complex V). Complexes I–IV transfer reducing equivalents which are the products of the citric acid cycle and β-oxidation and pass them to molecular O_2_ to form water. This transfer of electrons is associated with a free energy change which is used by complexes I, III and IV to translocate protons from the matrix to the intermembrane space, and an electrochemical gradient is established (Chance and Mela, 1966). The ATP synthase creates a hydrophilic pathway across the inner mitochondrial membrane which allows protons to flow down their electrochemical gradient. As protons move through the ATP synthase they are used to drive the energetically unfavourable reaction between ADP and P*_i_* to form ATP (Alberts et al., 1998).

Mitochondria contain their own genome (mtDNA) which in humans is a circular, double stranded, ∼16.6 kb molecule present in multiple copies within an individual cell. MtDNA encodes 13 essential polypeptides of the OXPHOS system as well as 22tRNAs and 2 rRNAs (Anderson et al., 1981) giving the mitochondria their own protein synthetic system. Mutations, arising either by genetic or environmental insult, can affect all copies of the mitochondrial genome within a cell (homoplasmy) or a cell can contain a mixture of wild type and mutated mtDNA (heteroplasmy) (Taylor and Turnbull, 2005). In the presence of heteroplasmy a biochemical defect is observed only when a critical threshold of mutated mtDNA to wild-type mtDNA is reached. The mechanism by which clonal expansion of mutated mtDNA to high levels occurs is unknown. A recent study showed that molecules with large scale deletions, which are therefore smaller molecules, accumulate in mouse cortical neurones faster than smaller deletions, and probably wild-type molecules (Fukui and Moraes, 2009). Whilst this may explain the clonal expansion of individual mtDNA deletions with age, it does not explain the expansion of mtDNA point mutations as these molecules are identical in size to wild-type molecules. One theory is that clonal expansion occurs by random genetic drift (Elson et al., 2001). Unlike nuclear DNA, mitochondrial DNA is replicated independently of the cell cycle and not all molecules are always replicated (relaxed replication (Bogenhagen and Clayton, 1977)). This model suggests that relaxed replication of mtDNA coupled with random degradation of some mtDNA molecules can lead, through random intracellular drift, to one mutant genotype becoming the dominant genotype of the cell.

Acquired mtDNA mutations have been proposed to play an important role in ageing (Miquel et al., 1980) and mtDNA mutations have been shown to accumulate in a number of ageing human tissues (Brierley, 1997; Brierley et al., 1998; Cottrell et al., 2001a,b; Muller-Hocker, 1989, 1990; Muller-Hocker et al., 1992). When mtDNA mutations clonally expand to a critical threshold level they may lead to respiratory deficiency which is demonstrated by the presence of cells which are cytochrome *c* oxidase (COX) deficient. The first documentation of the age-accumulation of COX deficient cells was by Muller-Hocker in 1989 who showed that COX deficient cardiomyocytes were not detectable before the age of 20, but were detected in all subjects examined above the age of 60 (Muller-Hocker, 1989). COX deficient cells have since been detected in a number of ageing tissues such as skeletal muscle (Muller-Hocker, 1990), various neuronal cell types (Cottrell et al., 2001a,b), colon (Taylor et al., 2003), stomach (McDonald et al., 2008) and liver (Fellous et al., 2009). However the contribution of these cells to the ageing process remains controversial. A mouse model with defective mitochondrial polymerase was developed to attempt to address the role of mtDNA mutations in ageing (Kujoth et al., 2005; Trifunovic et al., 2004). These mice have an increase in the frequency of mtDNA mutations compared to wild-type mice and display a number of ageing phenotypes, e.g., hair loss, kyphosis, reduced subcutaneous fat and osteoporosis, as well as a significantly reduced lifespan (Kujoth et al., 2005; Trifunovic et al., 2004; Vermulst et al., 2007). However they begin to accumulate mutations during embryonic development and it is unknown whether this occurs in humans. In addition a uniform distribution of mutation frequency between tissue types is observed which does not occur either in patients with mtDNA disease (Chinnery and Turnbull, 1999) or during normal human ageing (Krishnan et al., 2007). Therefore the relevance of these models to normal human ageing is unclear.

Studies on the role of mtDNA mutations in human ageing have tended to focus on post-mitotic tissues such as heart, muscle and neurons, whereas our recent studies have concentrated on replicating tissues with a particular emphasis on possible stem cell involvement. Human colon crypts provide an informative system in which to study the potential impact of mtDNA mutations in stem cell populations because the stem cell progeny are followed readily as they migrate up the crypt before being lost into the gut lumen. Thus crypts reflect the genotype of the stem cells at the base of the crypt. We have shown that mtDNA point mutations accumulate in human colonic stem cells with age, and that they expand clonally to high levels (Greaves et al., 2006; Taylor et al., 2003). Previously we have used a well-established technique to detect respiratory chain deficiency due to mtDNA mutations: the identification of cells which are deficient in COX but have normal succinate dehydrogenase (SDH) activity (Old and Johnson, 1989). COX contains subunits encoded by both the mitochondrial and nuclear genomes, whilst SDH is the catalytic part of complex II and is entirely nuclear encoded. However, since COX is only one of 4 respiratory chain complexes, it is possible that the impact of clonally expanded mtDNA mutations in human tissues with increasing age is much greater than is apparent from the study of COX alone. Complexes I and III of the respiratory chain also contain mtDNA encoded subunits but no histochemical techniques are available to investigate complex I and III activity. However antibodies specific to subunits of these complexes are commercially available and these have been shown to detect a biochemical deficiency in patients with mtDNA disease (Hanson et al., 2002). To investigate the true extent of respiratory chain deficiency in human colon due to mtDNA mutations, we used monoclonal antibodies to individual subunits of each of the four respiratory chain complexes to investigate expression at the protein level and to determine COX activity by histochemistry within the same crypts. In addition, we investigated the molecular genetic defects present in crypts with respiratory chain deficiency.

## 2 Materials and methods

### 2.1 Patients and colonic samples

Colonic mucosal samples were collected from two sources. Subjects (10) undergoing colon resection for colon cancer (age range 35–84 years); each sample of normal colonic mucosa was collected at a distance of at least 12 cm from the edge of the tumour. Mucosal biopsies from patients (10) undergoing colonoscopy for a disturbance of bowel function in whom no bowel pathology was identified (age range 18–80 years). All persons gave informed consent prior to inclusion in the study and ethical approval was obtained by the Joint Ethics Committee of Newcastle and North Tyneside Health Authority and the Northumberland Local Research Ethics Committee.

### 2.2 Cytochrome *c* oxidase/succinate dehydrogenase histochemistry and DNA isolation from single cells

Colon samples were mounted for sectioning and frozen in isopentane previously cooled to −190 °C in liquid nitrogen. Cryostat sections (12 μm) were cut onto glass slides and incubated in COX (100 μM cytochrome *c*, 4 mM diaminobenzidine tetrahydrochloride and 20 μg.ml^−1^ catalase in 0.2 M phosphate buffer pH 7.0) at 37 °C for 50 min. Sections were then washed in phosphate buffered saline (3 × 5 min) and then incubated in SDH medium (130 mM sodium succinate, 200 μM phenazine methosulphate, 1 mM sodium azide, 1.5 mM nitroblue tetrazolium in 0.2 M phosphate buffer pH 7.0) at 37 °C for 45 min. Sections were washed in phosphate buffered saline, pH 7.4 (3 × 5 min), dehydrated in a graded ethanol series (70%, 95%, 2 × 100%), cleared in Histoclear® (National Diagnostics, Atlanta, USA) and mounted in DPX. For laser microdissection, 20 μm cryostat sections were mounted on PEN (polyethylenenaphthalate) membrane slides (Leica Microsystems), subjected to sequential COX/SDH histochemistry as above and air-dried after ethanol dehydration. Single colonocytes were cut into sterile 0.5 ml PCR tubes using a Leica Laser Microdissection (AS-LMD) System, and lysed in standard lysis buffer (Taylor et al., 2003).

### 2.3 Sequencing of individual colonocytes

The entire sequence of the mitochondrial genome from microdissected colonocytes was determined using the single cell lysate (see above) as the DNA template. Cells underwent two rounds of PCR as previously described (Taylor et al., 2001). PCR products were cycle sequenced using ABI BigDye chemistries per standard manufacturer’s protocols and anaylsed on an ABI3100 genetic analyser (Applied Biosystems). Sequences obtained were compared to the rCRS and the homogenate sequence for that patient, using SeqScape software (Applied Biosystems).

### 2.4 Mitochondrial complex subunit immunohistochemistry

Serial 10 μm transverse sections were cut and allowed to air dry for 1 h at room temperature. They were then fixed in 4% paraformaldehyde in 0.1 M phosphate buffer for 10 min at 4 °C and then rinsed in distilled water. The sections were then permeabilised in a graded methanol series (70%, 95%, 100% v/v) over a period of 1 h. Endogenous peroxidase activity was quenched by the addition of 0.3% (v/v) hydrogen peroxide to the 95% methanol. The sections were than rinsed in PBS containing 0.1% Triton-X for 5 min, then incubated with antibodies to individual subunits of each of the oxidative phosphorylation enzyme complexes I–IV (complex I NDUFS3 10 μg/ml, complex II FeS 5 μg/ml, complex III Core II 2 μg/ml, complex IV subunit 1 5 μg/ml, Mitosciences) diluted in 4% BSA in PBST, for 1 h at room temperature. They were then washed 3 × 5 min in PBST followed by incubation with a peroxidase-conjugated antibody (rabbit anti-mouse Ig’s, 50 μg/ml; DAKO Ltd., Ely UK) for 1 h at room temperature. Following 3 × 5 min washes in PBST, peroxidase activity was demonstrated by incubating in 1.5 mM 3,3′-diaminobenzidine tetrahydrochloride and 0.01% (v/v) hydrogen peroxide in 0.1 M phosphate buffer pH 7.4, for 5 min at room temperature. Sections were then washed in distilled water, counterstained in Meyers Haemalum for 20 s and washed in running tap water. They were then dehydrated in a graded ethanol series (70%, 95%, 100%), cleared in Histoclear (National Diagnostics, Atlanta, Georgia, USA) and mounted in DPX (BDH laboratory supplies, Poole, UK). A densitometric analysis programme (KS300, Axiovision) was used to assess the protein expression levels. A crypt was defined as deficient for any of the complex subunits if the intensity of staining was 50% or less than that of positive crypts within the same subject.

## 3 Results

**Fig. 1:**
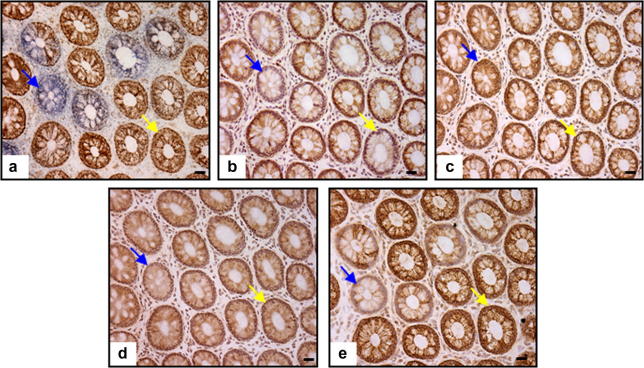
Respiratory chain deficiency in human colonic crypts. Example panel of serial transverse crypt sections which have undergone (a) COX/SDH histochemistry (crypts which are brown have COX activity, those which are blue have an absence of COX activity) and (b–e) immunohistochemistry to subunits of complexes I–IV, respectively (scale bars 20 μm). The blue arrows show a crypt with reduced expression of subunits of complexes I, III and IV. The yellow arrows show a crypt with an expression defect in complex I only.

**Fig. 2:**
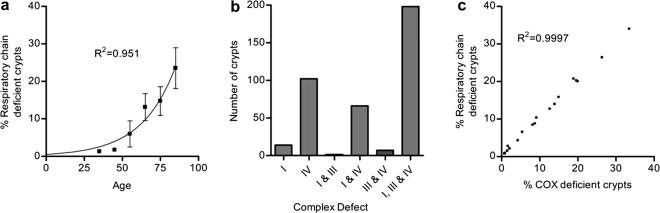
(a) Frequency of respiratory chain deficiency in human colonic crypts versus age. Respiratory chain deficiency defined as any crypt showing down regulation of any of the subunits of any complex. Subjects are grouped by decade, error bars show standard error of the mean (SEM). (b) The frequency of individual crypts with various degrees of respiratory chain deficiency. (c) Correlation between COX deficiency and total respiratory chain deficiency.

Serial transverse colon sections were cut from mucosal biopsies from 20 patients (aged 18–84 years) and histochemistry and immunohistochemistry performed. A total of 3471 crypts were examined for the expression levels of subunits of respiratory chain complexes I–IV. Fig. 1 shows an example panel of serial sections. We observed that 11.2% (388 crypts) had absence, or reduced expression, of one or more respiratory chain complex subunits. Respiratory chain deficiency increased exponentially with age (Fig. 2a) in a pattern identical with that observed in our previous studies of COX deficiency in individual crypts (Taylor et al., 2003). Of the 11% of crypts with decreased expression of respiratory chain subunits, 29.9% (116 crypts) had a decrease in only one complex; 19.1% (74 crypts) in two complexes, and 51% (198 crypts) in three complexes (I, III and IV) (Fig. 2b). Although isolated reduction of expression of complex I and complex IV subunits occurred, we did not observe isolated complex III deficiency. Surprisingly, we observed more isolated complex IV deficiency than isolated complex I deficiency. Complex IV subunit I expression was down-regulated in all crypts which showed no COX activity. It was also reduced in 94.3% of the crypts which showed any respiratory chain expression defects and there was a very close correlation (*R*^2^ = 0.997) between percentage COX activity deficiency and percentage crypts with decreased respiratory chain subunit expression (Fig. 2c).

Next we investigated the potential mitochondrial genetic basis for the observed decreased expression of respiratory chain subunits. Due to the recessive nature of mtDNA mutations and the fact that there are multiple copies of the mitochondrial genome present within individual cells, a mtDNA mutation must expand clonally to high levels before it causes a detectable biochemical defect. We have shown previously that mtDNA mutations clonally expand with age and that the frequency of these mutations increases with age (Taylor et al., 2003). However we have investigated previously only cells which are COX positive or COX deficient based on activity. In the present study we extended this analysis to examine cells that had defective expression of either single or multiple complexes to investigate the types of mtDNA mutation which are potentially pathogenic. We isolated, by laser microdissection, 2 cells with complex I deficiency only and 16 cells with combined complex I, III and IV deficiencies and sequenced the entire mitochondrial genomes. Due to potential problems with low mtDNA copy number PCR (Yao et al., 2007), all putative mutations were re-amplified from the original DNA lysate and were sequenced in both the forward and reverse directions. This strategy aimed to eliminate any errors due to either amplification of nuclear pseudogenes or errors introduced by the DNA polymerase during PCR amplification.

**Fig. 3:**
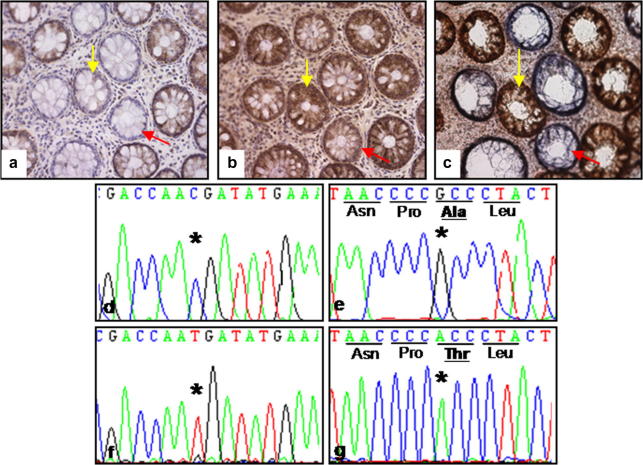
Mutations in cells isolated from colonic crypts with single and multiple complex expression defects. Serial transverse sections which have undergone immunohistochemistry for complexes I (a) and III (b), and COX/SDH histochemistry (c). The yellow arrow shows a crypt with isolated complex I deficiency, the red arrow shows a crypt which has combined complex I, III and IV deficiency. (d) Electropherogram showing the 14704T>C transition found in the crypt with multiple complex deficiency. This mutation is in the gene encoding the mitochondrial tRNA for glutamic acid (*MT-TE*). (e) Sequencing electropherogram showing the 13681A>G transition a crypt with isolated complex I deficiency. This change predicts a threonine to alanine amino acid change at position 449 of *MT-ND5*. Wild type homogenate sequences are shown in panels (f) and (g).

A wide variety of mutations were detected (Table 1). Fig. 3a–c shows an example of cells isolated from crypts which had reduced expression of the complex I subunit only, and cells from crypts which had decreased expression of subunits of complexes I, III and IV combined. The electropherograms showing the mutations detected in those cells are shown in Fig. 3d–g. In the two cells with isolated complex I defects we detected two homoplasmic mutations in complex I genes. The first, an m.10971G>A transition, predicts a tryptophan to termination codon substitution at position 71 (p.W71X) within NADH Dehydrogenase 4 encoded by the *MT-ND4* gene, predicting the premature truncation of the protein. The second, an m.13681A>G transition, predicts a p.T449A mutation in NADH Dehydrogenase 5 (encoded by the *MT-ND5* gene) (Fig. 3e). This mutation has been reported previously as a polymorphic variant (Brandon et al., 2005) and it is uncertain that this is causal for the complex I defect in this cell. The same mutation was also detected in a cell with multiple complex expression defects but this cell contained a second change m.2559T>G in the *MTRNR-2* gene which we predict is the likely cause of the multiple respiratory chain complex defects. In most cells, we detected a single clonally expanded mtDNA point mutation only.

A number of different changes in both protein encoding genes and RNA genes (see example electropherogram in Fig. 3d) were found in cells with multiple complex expression defects (Table 1). All of the mutations found in protein encoding genes predicted amino acid changes and all but one were present at levels >50%, suggesting that they are pathogenic. There did not appear to be a particular pattern of mutations, e.g., there was not a higher proportion of mutations in one gene compared with the rest, and the types of changes observed were similar to those which we have detected previously in COX deficient human colonic crypts (Greaves et al., 2006; Taylor et al., 2003). We were unable to detect mutations in 5 of the 18 cells sequenced.

## 4 Discussion

In this investigation we have again demonstrated that respiratory chain deficient crypts accumulate in human colon with age (Taylor et al., 2003). Furthermore, we have shown that multiple complexes of the respiratory chain are involved in more than 50% of deficient crypts. We have also shown that clonally expanded point mutations of the mtDNA are present in crypts with reduced expression of multiple complex subunits. Our previous studies examined crypts using an assay which detects COX deficiency only and in these studies we showed that the point mutations present in COX deficient crypts were not necessarily in COX encoding genes. This suggests that it is likely that multiple complexes were also affected in those crypts.

Our current data show that a point mutation is potentially able to disrupt the entire respiratory chain; this raises questions as to the mechanisms by which this may occur. A mutation in one of the tRNA or rRNA encoding genes will impair protein translation of some or all of the mtDNA encoded subunits of the respiratory chain (Jacobs, 2003). Indeed patients with mutations in mitochondrial rRNA and tRNA genes have multiple complex deficiencies (Hao and Moraes, 1996; Hinttala et al., 2006). However this does not explain how mutations in protein encoding genes affect multiple complexes. There is some evidence that the absence of one complex can affect the stability of another. In cultured mammalian cells with a known mutation in the cytochrome *b* subunit of complex III, steady state levels of both complex I and complex III are reduced (Acin-Perez et al., 2004). However in cell lines with a mutation in the ND6 subunit of complex I, complex III remains normal, suggesting that complex I is dependent on complex III for its stabilisation but complex III is stable in the absence of complex I (Acin-Perez et al., 2004). Our study detected crypts deficient in complex I alone, but no crypts which were deficient in complex III alone which supports this hypothesis. Further, multiple complex defects have been observed in patients with mutations in structural subunits of complex I (Hinttala et al., 2006) and complex III (Bruno et al., 2003; Rana et al., 2000).

It has been proposed that the complexes of the respiratory chain do not exist simply as individual complexes within the inner mitochondrial membrane but that they associate to form ‘supercomplexes’ (Schagger and Pfeiffer, 2000) or ‘respirasomes’ (Acin-Perez et al., 2008). Recent data has shown that these supercomplexes are able to respire when isolated by blue native gel electrophoresis (BNGE) and by the same method it was demonstrated that supercomplexes are not formed if one of their component complexes is absent (Acin-Perez et al., 2008). A recent study which modelled mutations in structural subunits of COX in a *Rhodobacter sphaeroides* model system reported that one of the mutations caused an intrinsic proton leak, which is predicted to affect the whole of the respiratory chain (Namslauer and Brzezinski, 2009). This provides further evidence that the complexes interact very closely and a defect of one complex may affect the stability of other complexes.

In five of the cells sequenced we were unable to detect a clonally expanded mtDNA point mutation. We do not know what causes the respiratory chain defect in these cells, but there are a number of possible explanations. We have previously searched for large scale mtDNA deletions in human colonocytes without success (Taylor et al., 2003) and on this basis we excluded the possibility that deletions are the cause of the respiratory chain defects. There may be multiple different mutations present at low levels in these cells which act together to produce the biochemical defect (Trifunovic and Larsson, 2008), or there may be dominant mtDNA mutations which cause a biochemical defect at low mutation levels (Sacconi et al., 2008). It is also possible that nuclear encoded genes involved in mitochondrial protein import or assembly may cause respiratory chain defects (Papadopoulou et al., 1999; Sue et al., 2000).

We have shown that respiratory chain deficiency in the human colon has a strong positive association with age. We have also demonstrated for the first time that multiple complexes of the respiratory chain are affected simultaneously and that the COX/SDH histochemical assay is an extremely effective method to identify cells which are respiratory chain deficient and contain mtDNA mutations. These studies also highlight the prevalence of this phenomenon in an ageing stem cell population. Since clonally expanded mtDNA mutations are also present in other human stem cell populations (McDonald et al., 2008; Nekhaeva et al., 2002), it is important to consider the impact of these changes on the human ageing process, and further studies are required to investigate this. Finally we have shown that human colonic mucosal crypts are a highly informative model system for our understanding of mitochondrial genetics and mitochondrial function.

## Figures and Tables

**Table 1:** Mutations found in cells taken from crypts with single and multiple complex deficiencies.

Subject	Cell	Complex expression defect	Mutation	Gene	Level (%)	Amino acid change	Amino acid conservation	Database status
1	1	I,III,IV	m.13681A>G	*MT-ND5*	100	p.T449A	Poor	mtDB: 10/2704
1	1	I,III,IV	m.2559A>G	*MT-RNR2*	50	–	–	Not reported
1	3	I,III,IV	m.9714G>A	*MT-CO3*	100	p.G170S	Moderate	Not reported
1	5	I,III,IV	m.5997G>A	*MT-CO1*	100	p.A32T	Poor	Not reported
1	6	I,III,IV	m.1905G>A	*MT-RNR2*	100	–	–	Not reported
1	7	I,III,IV	m.14704T>C	*MT-TE*	100	–	–	Not reported
1	8	I only	m.13681A>G	*MT-ND5*	100	p.T449A	Poor	mtDB: 10/2704
2	1	I,III,IV	m.10761T>C	*MT-ND4L*	80	p.C98R	High	Not reported
2	2	I,III,IV	m.1101 A>G	*MT-ND4*	50	p.S86G	Poor	mtDB: 1/2704
2	3	I,III,IV	m.9247G>A	*MT-CO3*	100	p.S14 N	High	Not reported
2	4	I,III,IV	m.15077G>A	*MT-CYB*	100	p.E111L	Moderate	mtDB: 2/2704
2	8	I,III,IV	m.13916G>A	*MT-ND5*	40	p.G526E	High	Not reported
2	9	I,III,IV	m.2816G>A	*MT-RNR2*	100	–	–	Not reported
2	10	I only	m.10971G>A	*MT-ND4*	100	p.W71X	High	Not reported

Single cells were laser-microdissected from colonic crypts with expression defects in single and multiple complexes, and their entire mitochondrial genome sequenced. The changes listed here are not present in the homogenate mtDNA sequence for that subject. Amino acid conservation was assessed using the PIR-International Protein Sequence Database, (Wu et al., 2002). *Abbreviations:* T, threonine; A, alanine; G, glycine; S, serine; C, cysteine; R, arginine; N, asparagine; E, glutamic acid; L, lysine; W, tryptophan; TERM, termination codon. The following databases were checked to determine whether these had previously been reported as mtDNA polymorphisms: (i) MITOMAP (http://www.mitomap.org/), (Brandon et al., 2005a); (ii) mtDB (http://www.genpat.uu.se/mtDB/), (Ingman and Gyllensten, 2006).
